# Surveillance of recent HIV infections among newly diagnosed HIV cases in Germany between 2008 and 2014

**DOI:** 10.1186/s12879-017-2585-4

**Published:** 2017-07-11

**Authors:** Alexandra Hofmann, Andrea Hauser, Ruth Zimmermann, Claudia Santos-Hövener, Jörg Bätzing-Feigenbaum, Stephan Wildner, Claudia Kücherer, Norbert Bannert, Osamah Hamouda, Viviane Bremer, Barbara Bartmeyer

**Affiliations:** 1 Department for Infectious Disease Epidemiology, Unit 34 HIV/AIDS, STI and Blood-Borne Infections, Robert Koch-Institute, Seestr.10, 13353 Berlin, Germany; 2 Charité, Universitätsmedizin, Berlin, Germany; 3 Department of Infectious Diseases, Unit 18 HIV and Other Retroviruses, Robert Koch-Institute, Nordufer 20, 13353 Berlin, Germany; 4 Central Research Institute of Ambulatory Health Care in Germany (Zi), Berlin, Germany; 5 SGS Belgium NV, Generaal De Wittelaan 19, B-2800 Mechelen, Belgium; 6 Department for Infectious Disease Epidemiology, Robert Koch-Institute, Seestr.10, 13353 Berlin, Germany

**Keywords:** Surveillance, HIV, Recent HIV infection, Test for recency of infection, Bed-CEIA, Germany

## Abstract

**Background:**

The HIV surveillance system in Germany is based on mandatory, anonymous notification of newly diagnosed HIV cases by laboratories. Because the time between HIV infection and the diagnosis of HIV varies widely between persons, it is difficult to determine the number of cases of recent HIV infection among newly diagnosed cases of HIV. In Germany, the BED-capture-enzyme immunoassay (BED-CEIA) has been used to distinguish between recent and long-standing HIV infection. The aim of this analysis is to report the proportion of cases of recent HIV infection among newly diagnosed cases in Germany between 2008 and 2014 and to identify factors associated with recent infections.

**Methods:**

A sample of voluntary laboratories among all HIV diagnostic laboratories was recruited. Residual blood from HIV diagnostic tests was spotted on filter paper as dried serum or dried plasma spots and was sent along with the notification form of the HIV cases. The BED-CEIA test was performed. A case was defined as recent HIV infection with a BED-CEIA test result of less than 0.8 normalized optical density, with the exclusion of CDC stage C. The proportion of recent newly diagnosed HIV infections among different groups (such as transmission groups, gender or age groups) was calculated. We used logistic regression to identify factors associated with recent HIV infection and to identify subpopulations with high proportions of recent HIV infections.

**Results:**

Approximately 10,257 newly diagnosed cases were tested for recency using the BED-CEIA. In total, 3084 (30.4%) of those were recently infected with HIV. The highest proportion of recent HIV infections was found among men who had sex with men (MSM) (35%) and persons between 18 and 25 years of age (43.0%). Logistic regression revealed that female German intravenous drug users with a recent HIV infection had a higher chance of being detected than German MSM (OR 2.27).

**Conclusions:**

Surveillance of recent HIV infection is a useful additional tool to monitor the HIV epidemic in Germany. We could observe ongoing HIV transmission in Germany in general and in different subgroups, and we could identify factors associated with recent HIV infection in Germany.

## Background

In 2014, 83,400 (77,000–91,200) people were estimated to be living with HIV/AIDS in Germany. The majority of them were men who have sex with men (MSM; approximately 53,800), followed by people who were infected heterosexually with HIV (HET; approximately 10,500) and approximately 7900 persons who were intravenous drug users (IDU). The estimated HIV epidemic in Germany peaked in 1985, followed by a decline in the 1990s, an increase from 2000 to 2006, and stable infection rates from 2006 to 2014. It was estimated by modelling that in 2014, approximately 3200 people were newly infected with HIV. The highest estimated number of HIV infections occurred among MSM (2300) [1].

In Germany, the HIV surveillance system is based on mandatory, anonymous notification of newly diagnosed HIV cases by laboratories. Additional epidemiological information regarding the HIV mode of transmission and other clinical data is reported by physicians. AIDS case reporting is voluntary [2]. In addition to HIV notification data, several long-term observational cohort studies in different HIV-positive populations, such as the study on the clinical surveillance of HIV disease [3], the German HIV seroconverter study [4] and other biological and behavioural studies in the most affected vulnerable groups have been performed.

The time between HIV infection and the diagnosis of HIV varies widely between individuals. Therefore, it is difficult to calculate the number of persons newly infected with HIV per year to determine the incidence of HIV infections by exclusively using newly diagnosed HIV cases. As a consequence, all other supportive sources, such as the HIV cohorts and the mortality register, are used to model the incidence of new HIV infections per year and the number of people who live with HIV/AIDS in Germany by using the imputation method [1, 5].

In addition to modelling an estimated incidence and prevalence of HIV infected persons in Germany, cases of newly diagnosed HIV are regularly tested serologically for recent HIV infection (TRI) since 2011 to improve the method of estimating the HIV epidemic in Germany and to identify populations with recent transmission of HIV. Numerous serological assays for TRI were developed and applied within the last decade [6–9]. Most of the tests are based on the measurement of the maturation of HIV-1 specific antibodies, which occur within the first two years after seroconversion [10, 11]. Accordingly, based on the increase of the antibody titres [12–14], the proportion of HIV-specific immunoglobulin G (IgG) antibodies relative to total IgG [15] or the avidity of antibodies [16–20], TRIs can distinguish between recently acquired and long-standing infections. For many years, the BED-capture-enzyme immunoassay (BED-CEIA) [15] was the most commonly used recency assay [21–23]. The BED-CEIA was evaluated for Germany in a pilot study (2005–2007) [24] and finally applied in a nationwide cross-sectional incidence study [25, 26]. The findings in this study were used to implement a nationwide surveillance system of recent HIV infections in 2011.

The aim of this analysis is to report the proportions of recent HIV infections among newly diagnosed HIV cases in Germany between 2008 and 2014 and to identify factors associated with recent infection in order to provide insight into current HIV transmission dynamics.

## Methods

### Sample collection

**Fig. 1:**
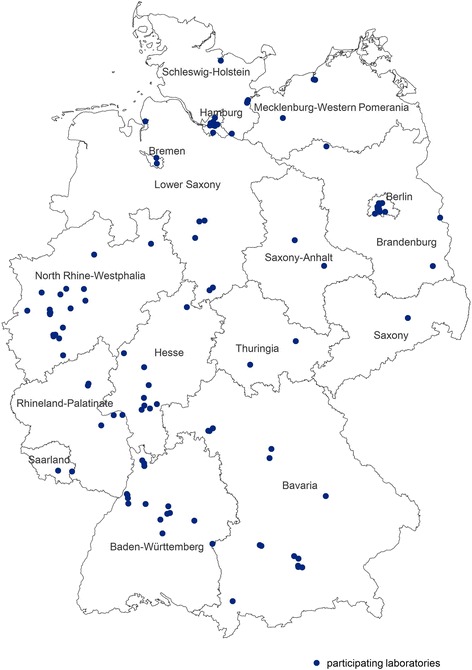
Distribution of participating laboratories (*n* = 108) in Germany, 2008–2014. Map created with RegioGraph Analyse © GfK GeoMarketing GmbH, Bruchsal, Germany

Newly diagnosed HIV cases were directly reported by laboratories to the Robert Koch Institute, RKI, which is the national public health institute of Germany. As the number of HIV notifications varies a lot between the reporting laboratories in Germany, only a subgroup of laboratories were recruited using convenience sampling for this analysis (*n* = 108; Fig. 1). This subgroup of laboratories notified 90.5% of all newly HIV diagnosed cases between 2008 and 2014. These laboratories collected blood residuals from HIV diagnostics, which were spotted on filter paper (Whatman # 903, GE Healthcare Bio-Science Corp, Westborough, USA) as dried serum spots (DSS) or dried plasma spots (DPS) for the surveillance of recent HIV infections. The DSS/DPS samples were sent together with the routine HIV notification form to the RKI. For this analysis, DSS/DPS samples were used from two different studies. They were collected between 1st March 2008 and 30th June 2010 for a nationwide cross-sectional study and between the 1st January 2011 and 31st December 2014 for the study “surveillance of recent HIV infections”.

**Fig. 2:**
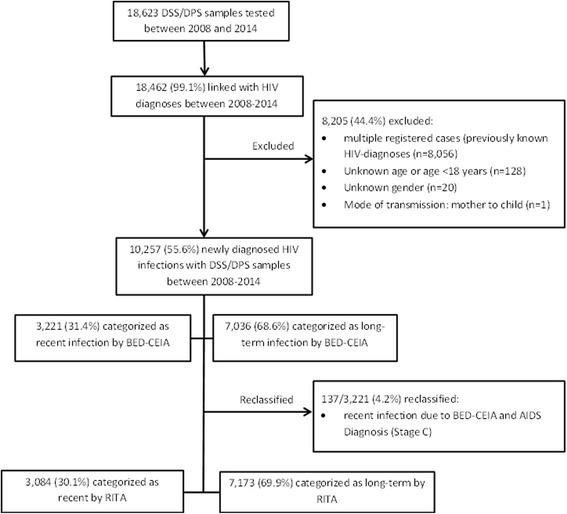
Flowchart of DSS/DPS samples and HIV diagnoses included in the analyses between 2008 and 2014 in Germany. DSS/DPS: dried serum spots or dried plasma spots. RITA: recent test algorithm. BED-CEIA: BED-capture-enzyme immunoassay

For this analysis, only persons were included, who were newly diagnosed with HIV between 2008 and 2014 and with one additional DSS/DPS sample available. HIV notifications and DSS/DPS samples were linked by using the number of the HIV notification form. Data on age, gender, mode of transmission, region of origin and assumed region of infection were used from the associated HIV notification form. Exclusion criteria for this analysis were unknown information on gender or age, age below 18 years, being infected with HIV via mother to child transmission, or DSS/DPS samples from double-notified HIV cases (Fig. 2). Ethical approval for the nationwide cross-sectional study was given by the ethics board at the Charité, University Medicine, Berlin, as well as approval from the data protection office of Germany according to the Federal Data Protection Act. The DSS/DPS samples used were residuals from routine HIV diagnostic processing; therefore, no patient informed consent was given. Furthermore, the DSS/DPS samples cannot be linked to an individual person because the HIV notification system is strictly anonymous. Additionally, the BED-CEIA is only licensed for epidemiological studies and not for individual diagnosis. Therefore, no extra benefit can be obtained by informing persons about positive BED-CEIA test results.

### Laboratory testing

All DSS/DPS samples were tested for recency of HIV infection by the RKI study laboratory (HIV and Other Retroviruses Unit) using the BED-CEIA from the Calypte Biomedical Corporation, Portland, USA [27], and since 2014, the BED-CEIA from Sedia Biosciences Corporation, Portland, USA was used [28]. The BED-CEIA measures the proportion of HIV-1-specific immunoglobulin G (IgG) antibodies relative to total IgG at a normalized optical density (ODn). Cases were classified as recent HIV infection or long-standing infection by the quantitative output relative to a defined cut off of ODn <0.8. A recent HIV infection is determined, according to Sedia Biosciences Corporation, as an infection that occurred within the last 197 (range 127–236) days, or within the last 162 days for HIV subtype B [28]. Calypte reports a duration of 155 days [27], and in Germany, approximately 20 weeks (=140 days) was determined as the duration of a recent HIV infection [29]. The sensitivity and specificity of the BED-CEIA are low (81.7%; 89.1%) [15], and therefore, the test is recommended for surveillance purposes only using DSS/DPS samples from individuals who have already been diagnosed with HIV.

To minimize false recent classification of the BED-CEIA, an additional step, the recent test algorithm (RITA), was applied following the European Centre for Disease Prevention and Control (ECDC) recommendation [30]. It is recommended that cases with a recent BED-CEIA test result should be recoded as long-standing infection if the case was reported with an AIDS defining illness (stage C of the Centers for Disease Control and Prevention (CDC) criteria), CD4 cell counts < 200 cells/mm^3^_,_ or a reported plasma viral load below 400 copies/ml. Data on CD4 cell counts and viral load are often missing in the HIV notification form and are therefore scarce. For example, in 2014, only 34% of the newly diagnosed HIV cases had information regarding CD4 cell count [31]. Therefore, only the information about AIDS defining illnesses reported on the notification form was used to minimize false BED-CEIA test results.

A newly diagnosed case of recent infection with HIV is therefore defined as a BED-CEIA test result with a cut off of less than 0.8 ODn and without any documented AIDS defining illnesses. In contrast, a case with long-standing HIV infection is defined as a case in which the BED-CEIA test result has a cut off equal to or higher than 0.8 ODn or an AIDS defining illness. By using this method, cases with a false recent test result can be identified and corrected; however false long-standing (FLS) test results were not corrected. To correct the FLS test results, as well, further information about the cases is needed, such as previously documented HIV RNA+/Ab- or p24Ag+/Ab- within 180 days of the sample date, clinical symptoms of seroconversion, or previously documented HIV-negative test result within 180 days of the sample date [30]. This information is not available on the HIV notification form.

### Statistical analysis

Analyses were performed using STATA 13.0 (Stata Statistical Software: Release 13, United States). To guarantee comparability between newly diagnosed HIV cases with DSS/DPS and all newly diagnosed HIV cases, a weight has been calculated by dividing the proportion of variable specifications among all newly diagnosed cases with the proportion of variable specifications of those newly diagnosed cases with DSS/DPS.

We analysed the number and the proportion of recent HIV infections among cases using the chi^2^ test for bivariate comparison and logistic regression to assess the odds ratio for recent infections. Univariate logistic regression was performed. Factors with significant odds ratios were included in the multivariable logistic regression. Time trends were analysed by using logistic regression with recent infection as a dependent variable and diagnosed cases with DSS/DPS per year as an independent variable. Multivariable logistic regression with recent infection as a dependent variable was also used to identify subgroups according to their gender and origin within different modes of transmission. Unknown data was included into analysis, such as unknown mode of transmission, origin or assumed region of infection for identifying subgroups. For the proportion of recent infections, the 95% confidence interval (95% CI) was calculated. A Mann-Whitney (Wilcoxon) rank sum test of medians was used to compare the age of those with recent infection and those with long-standing infection.

## Results

### Participating laboratories and availability of DSS/DPS among newly diagnosed HIV cases

Between 2008 and 2014, a total of 39,170 HIV diagnoses were reported by 227 laboratories nationwide. The participating laboratories (*n* = 108/227; 47.6%) reported approximately 91.3% (*n* = 35,756) of all HIV diagnoses between 2008 and 2014. Of these 39,170 HIV diagnoses, 20,896 (53.3%) were newly diagnosed cases (double or unknown notifications were excluded from analysis). During the same time period, a total of 18,623 DSS/DPS samples were sent to the RKI by these laboratories and 99.1% could be linked to an HIV notification form (Fig. 2). The distribution of the numbers of reported HIV cases by laboratories within subgroups is similar for newly diagnosed HIV cases with DSS/DPS (*n* = 10,257) and all newly diagnosed cases (*n* = 20,487 Table 1). The different weights ranged between 0.9 and 1.1 (Median 1), therefore no additional weight adjustment was included. Especially, the number of collected DSS/DPS samples differed in 2010 (*n* = 4.0), when DSS/DPS samples were collected for a shorter time period than in other years. The number of collected DSS/DPS samples increased significantly over time (chi^2^ *p* < 0.001).

**Table 1:** Comparison of newly diagnosed HIV cases with and without DSS/DPS samples by gender, origin, age, mode of transmission, region of presumed infection, region of origin and year of diagnosis, 2008–2014, Germany

	Newly diagnosed HIV cases, n %				
	total		with DSS/DPS^4^ Sample		weighting
Total	20,487	100%	10,257	100%	
Gender					
Men	17,137	83.6%	8,541	83.3%	1.0
Women	3,350	16.4%	1,716	16.7%	1.0
Region of origin					
Germany	12,830	62.6%	6,405	62.4%	1.0
Abroad	5,454	26.6%	2,853	27.8%	1.0
Unknown	2,203	10.8%	999	9.7%	1.1
Age group					
< 25 years	2,298	11.2%	1,216	11.9%	0.9
25–34 years	6,687	32.6%	3,443	33.6%	1.0
35–44 years	5,999	29.3%	2,986	29.1%	1.0
45–54 years	3,824	18.7%	1,838	17.9%	1.0
> =55 years	1,679	8.2%	774	7.5%	1.1
Mode of transmission					
MSM^1^	11,541	56.3%	5,993	58.4%	1.0
HET^2^	3,789	18.5%	1,909	18.6%	1.0
IDU^3^	649	3.2%	307	3.0%	1.1
Unknown	4,508	22.0%	2,048	20.0%	1.1
Region of presumed infection					
Germany	14,184	69.2%	7,124	69.5%	1.0
Abroad	3,506	17.1%	1,803	17.6%	1.0
Unknown	2,797	13.7%	1,330	13.0%	1.1
Year of Diagnosis					
2008	2,745	13.4%	1,264	12.3%	1.1
2009	2,815	13.7%	1,335	13.0%	1.1
2010^a^	2,653	12.9%	332	3.2%	4.0
2011	2,644	12.9%	1,624	15.8%	0.8
2012	2,920	14.3%	1,779	17.3%	0.8
2013	3,232	15.8%	1,890	18.4%	0.9
2014	3,478	17.0%	2,033	19.8%	0.9

^1^ *MSM* men who have sex with men

^2^ *IDU* persons who are intravenous drug users

^3^ *HET* persons with heterosexual contact

^4^ *DSS*/*DPS Sample* Dried Serum Spot/Dried Plasma Spot

^a^DSS/DPS samples were not continuously collected due to the end of the first study in June 2010

### Characteristics of the overall study population of newly diagnosed HIV cases with DSS/DPS samples between 2008 and 2014

The study population consisted mostly of men (83.3%; ﻿*n*﻿ =8,541/10,257). The main mode of HIV transmission was MSM (58.4%), followed by an unknown mode of transmission (20.0%), HET (18.6%) and IDU (3.0%). One third (33.6%) of the study population was between 25 and 34 years old (Table 2). Most patients originated from Germany (62.4%), followed by people from sub-Saharan Africa (9.5% (Table 3). The most frequently reported presumed region of infection was Germany (69.5%), followed by sub-Saharan Africa (7.2%) (Table 3).

**Table 2:** Characteristics and factors associated with recent HIV infection among newly diagnosed HIV cases using DSS/DPS samples in Germany, 2008–2014

	Newly diagnosed HIV cases, n %				Univariate analysis		Multivariable analysis (*n* = 10,257)	
Factor	total with DSS/DPS^4^		with recent infection		OR*	95% CI°	OR*	95% CI°
Total	10,257	100%	3,084	30.01%				
Gender								
Men	8,541	83.3%	2,705	31.7%	(ref)		(ref)	
Women	1,716	16.7%	379	22.1%	0.61#	0.54–0.69	0.94	0.79–1.12
Region of origin								
Germany	6,405	62.5%	2,121	33.1%	(ref)		(ref)	
Abroad	2,853	27.8%	673	23.6%	0.62#	0.56–0.69	0.74#	0.66–0.84
Unknown	999	9.7%	290	29.0%	0.83##	0.71–0.96	0.94	0.80–1.11
Age group								
< 25 years	1,216	11.9%	523	43.0%	(ref)		(ref)	
25–34 years	3,443	33.6%	1,091	31.7%	0.61#	0.54–0.70	0.63#	0.55–0.72
35–44 years	2,986	29.1%	846	28.3%	0.52#	0.46–0.60	0.53#	0.46–0.61
45–54 years	1,838	17.9%	470	25.6%	0.46#	0.39–0.53	0.45#	0.39–0.53
> =55 years	774	7.5%	154	19.9%	0.33#	0.27–0.41	0.35#	0.28–0.43
Mode of transmission								
MSM^1^	5,993	58.4	2,095	35.0%	(ref)		(ref)	
HET^2^	1,909	18.6	393	20.6%	0.48#	0.43–0.55	0.65#	0.54–0.79
IDU^3^	307	3.0	107	34.9%	1.00	0.78–1.27	1.18	0.92–1.51
Unknown	2,048	20.0	489	23.9%	0.58#	0.52–0.65	0.68#	0.60–0.77
Region of presumed infection								
Germany	7,124	69.4%	2,346	32.9%	(ref)		(ref)	
Abroad	1,803	17.6%	387	21.5%	0.56#	0.49–0.63	0.85##	0.73–0.99
Unknown	1,330	13.0	351	26.4%	0.73#	0.64–0.83	0.98	0.84–1.14

^1^MSM: men who have sex with men

^2^IDU: persons who are intravenous drug users

^3^HET: persons with heterosexual contact

^﻿4^ ﻿DSS/DPS: Dried Serum Spot/Dried Plasma Spot

^*^OR: odds ratio

^°^95% CI: 95% Confidence interval

# *p*-value: *p* < =0.001

## *p*-value: *p* < =0.05

**Table 3:** Origin and presumed region of infection in cases of recent HIV infection among newly diagnosed HIV cases using DSS/DPS samples in Germany, 2008–2014

	Newly diagnosed HIV cases, n %				Univariate analysis	
	Total with DSS/DPS^1^		With recent infection		OR*	95% CI°
Region of origin						
Germany	6,405	62.4%	2,121	33.1%	(ref)	
Eastern Europe	384	3.7	90	23.4%	0.62#	0.49–0.79
Western Europe	310	3.0	98	31.6%	0.93	0.73–1.19
Central Europe	464	4.5	143	30.8%	0.90	0.73–1.10
Asia / Oceania	274	2.7	65	23.7%	0.63#	0.47–0.83
America / Caribbean	265	2.6	68	25.7%	0.70##	0.53–0.92
North / Northeast Africa	90	0.9	20	22.2%	0.58##	0.35–0.95
sub-Saharan Africa	978	9.5	169	17.3	0.42#	0.35–0.50
Unknown region	88	0.9	20	22.7%	0.59##	0.36–0.98
Unknown	999	9.7	290	29.0%	0.83##	0.71–0.96
Region of origin (combined)						
Germany	6,405	62.4	2,121	33.1%	(ref)	
Abroad	2,853	27.8	673	23.6%	0.62#	0.56–0.69
Unknown	999	9.7	290	29.0%	0.83##	0.71–0.96
Region of presumed infection						
Germany	7,124	69.5	2,346	32.9%	(ref)	
Eastern Europe	215	2.1	41	19.1%	0.48#	0.34–0.68
Western Europe	205	2.0	70	34.1%	1.06	0.79–1.42
Central Europe	145	1.4	36	24.8%	0.67##	0.46–0.98
Asia / Oceania	266	2.6	54	20.3%	0.52#	0.39–0.70
America / Caribbean	128	1.2	30	23.4%	0.62##	0.41–0.94
North / Northeast Africa	43	0.4	13	30.2	0.88	0.46–1.70
sub-Saharan Africa	737	7.2	129	17.5%	0.43#	0.36–0.53
Unknown Region	64	0.6	14	21.9%	0.57	0.31–1.03
Unknown	1,330	13.0	351	26.4%	0.73#	0.63–0.83
Region of presumed infection (combined)						
Germany	7,124	69.5	2,346	32.9%	(ref)	
Abroad	1,803	17.6	387	21.5%	0.56#	0.49–0.63
Unknown	1,330	13.0	351	26.4%	0.73#	0.64–0.83

°95% CI: 95% confidence interval

*OR: odds ratio

^1^ ﻿DSS/DPS: Dried Serum Spot/Dried Plasma Spot﻿

# *p*-value: *p* < =0.001

## *p*-value: *p* < =0.05

Information about CD4 cell count among newly diagnosed HIV cases with DSS/DPS was available from 32.1% (*n* =3297). Of those cases, 51.6% showed CD4 cell counts below 350 cells (*n* = 1700). Late presentation (CD4 cell counts <350 cells/mm^3^ or CDC stadium AIDS) was identified in 10.1% (*n* =949/9363) of newly diagnosed cases with DSS/DPS with available data. Virus load information was available for 36.6% (*n* =3755/10,257) of newly diagnosed HIV cases with DSS/DPS.

### Proportion of recent HIV infections among newly diagnosed HIV cases with DSS/DPS samples between 2008 and 2014

In total, 3221 (31.4%; 95% CI 30.5–32.2%) cases were classified as recent infections, of which 137 (4.2%) were reclassified according to the ECDC recommendation (see Fig. 2) [30]. The final study population consisted of 30.1% of cases with recent infection (*n* = 3084; 95% CI 29.2–31.0%). The proportion of recent HIV infections among men was 31.7% compared to 22.1% among women (chi^2^ *p* < 0.001). The highest proportion of recent infections (43.0%) was found among young persons between 18 and 25 years of age (Table 2). Within the different modes of transmission, the highest proportion of recent infections was found among MSM (35.0%), followed by IDU (34.9%) (Table 2). Persons with a recent HIV infection were significantly younger than those with a long-standing HIV infection (median age 34 vs. 37 years; *p* < 0.001 Wilcoxon). MSM with recent HIV infection were significantly younger than MSM with a long-standing HIV infection (median age 33 vs. 36 years; *p* < 0.001 Wilcoxon). No difference in age regarding recent HIV infection could be found for HET (median age 35 vs. 36; *p* = 0.1215 Wilcoxon) and IDU (median age 34 vs. 36; *p* = 0.0990 Wilcoxon).

The proportion of recent infection was 33.1% among Germans and 17.3% among sub-Saharan Africans (Table 3). Newly diagnosed HIV cases from Germany were more likely to be recent infected than those identified among newly diagnosed HIV cases originating from abroad (33.1% vs. 23.6%; chi^2^ *p* < 0.001) (Table 2). Persons reported to be infected with HIV in the Western Europe region (excluding Germany) had the highest proportion of recent infections (34.1%), followed by people from Germany (32.9%) as the presumed region of infection (Table 3). Cases with a recent HIV infection were more likely infected with HIV in Germany than abroad (32.9% compared to 21.5%; chi^2^ *p* < 0.001). Cases from abroad with a recent infection were more likely reported to have acquired the infection in Germany (30.8% vs. 18.5%; chi^2^ *p* < 0.001). Cases with recent infection and available information on CD4 cell showed significantly higher CD4 cell counts than cases with long standing infection (Median 485 vs. 252 CD4 cells/mm^3^; *p* < 0.001 Wilcoxon). Among 2.2% (*n* =21/949) of cases defined as late presenters by CD4 cell count or CDC stage, a recent infection was identified by using the BED-CEIA.

### Proportion of recent infections over time within transmission groups

**Fig. 3:**
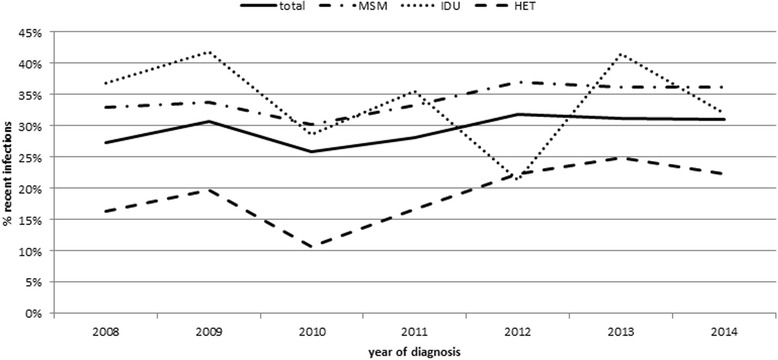
Proportion of recent HIV infections among newly diagnosed HIV cases using DSS/DPS samples in different transmission groups by year in Germany, 2008–2014. MSM: men who have sex with men. IDU: persons who are intravenous drug users. HET: persons with heterosexual contact

The newly diagnosed HIV cases with available DSS/DPS samples increased from 1264 in 2008 to 2033 in 2014. The DSS/DPS sample collection decreased in June 2010 due to the end of the first study (Table 1). The proportion of recent HIV infections per year varied from 26% in 2010 up to 32% in 2012, with an overall increase of 4% between 2008 and 2014 (data not shown). The overall increase could be confirmed by univariate logistic regression (OR 1.023; 95% CI 1.006–1.049; *p* < 0.012). A similar trend was observed for MSM (OR 1.030; 95% CI 1.003–1.058) and for HET (OR 1.079; 95% CI 1.02–1.140). No significant trend over time (OR 0.965; 95% CI 0.865–1.076; *p* < 0.521) was seen in IDU (Fig. 3).

### Association between recent infections and other variables

The univariate logistic regression revealed that several factors were associated with recent HIV infection, such as mode of transmission, age, gender and region of infection, which are shown in Table 2. These associations were confirmed by multivariable logistic regression, which showed that cases with a different mode of transmission than MSM were more likely to have a long-standing HIV infection. Including persons younger than 25 years, the proportion of persons with long-standing HIV infections increased with age (Table 2).

An additional multivariable logistic regression aimed to identify sub-groups within the main transmission groups revealed, that female German intravenous drug users had a higher chance of being diagnosed with a recent HIV infection (OR 2.49; 95% CI 1.40–4.41) than German MSM (Table 4). However, the numbers of female German drug users were small, with 28 (57.1%) cases of recent HIV infection among 49 female German drug users in total. Other groups with a lower chance of being recently infected with HIV compared to German MSM were German heterosexual females (OR 0.70; 95% CI 0.55–0.90), heterosexual females with an origin abroad (OR 0.44; 95% CI 0.35–0.54), heterosexual females with an unknown origin (OR 0.34; 95% CI 0.17–0.70) and heterosexual men with an origin abroad (OR 0.39, 95% CI 0.29–0.53) (Table 4).

**Table 4:** Characteristics of subpopulations within the different modes of transmission with recent HIV infection among newly diagnosed HIV cases with DSS/DPS samples in Germany, 2008–2014

Subpopulations			Newly diagnosed HIV cases, n %			Univariate analysis		Multivariable analysis (*n* = 10,257)	
			Total	With recent infection		OR*	95% CI°	OR*	95% CI°
MSM^1^	Men	German Origin	4,654	1,652	35.5%	(ref)		(ref)	
		non-German Origin	944	316	33.5%	0.91	0.79–1.06	0.87	0.75–1.02
		Unknown	395	127	32.2%	0.86	0.69–1.07	0.85	0.68–1.07
IDU^2^	Men	German Origin	92	37	40.2%	1.22	0.80–1.86	1.35	0.88–2.06
		non-German Origin	114	26	22.8%	0.54##	0.35–0.83	0.58##	0.37–0.91
		Unknown	24	7	29.2%	0.75	0.31–1.81	0.76	0.31–1.85
	Women	German Origin	49	28	57.1%	2.42##	1.37–4.28	2.49##	1.40–4.41
		non-German Origin	17	4	23.5%	0.56	0.18–1.72	0.52	0.17–1.61
		Unknown	11	5	45.5%	1.51	0.46–4.97	1.50	0.45–4.99
HET^3^	Men	German Origin	175	49	28.0%	0.71##	0.51–0.99	0.96	0.68–1.36
		non-German Origin	414	66	15.9%	0.34#	0.26–0.45	0.39#	0.29–0.53
		Unknown	13	6	46.2%	1.56	0.52–0.46	1.92	0.63–5.80
	Women	German Origin	347	95	27.4%	0.69##	0.54–0.87	0.70##	0.55–0.90
		non-German Origin	901	168	18.6%	0.42#	0.35–0.50	0.44#	0.35–0.54
		Unknown	59	9	15.3%	0.33##	0.16–0.67	0.34##	0.17–0.70
Unknown	Men	German Origin	960	236	24.6%	0.59#	0.51–0.69	0.67#	0.57–0.79
		non-German Origin	351	67	19.1%	0.43#	0.33–0.56	0.48#	0.36–0.63
		Unknown	405	116	28.6%	0.73##	0.58–0.91	0.80	0.62–1.03
	Women	German Origin	128	24	18.8%	0.42#	0.27–0.66	0.46#	0.30–0.73
		non-German Origin	112	26	23.2%	0.58##	0.35–0.86	0.58##	0.37–0.91
		Unknown	92	20	21.7%	0.50##	0.31–0.83	0.54##	0.32–0.90
Region of presumed infection									
Germany			7,124	2,346	32.9%	(ref)		(ref)	
Abroad			1,803	387	21.5%	0.56#	0.49–0.63	0.87	0.74–1.02
Unknown			1,330	351	26.4%	0.73#	0.64–0.83	0.97	0.83–1.14
Age group									
< 25 years			1,216	523	43.0%	(ref)		(ref)	
25–34 years			3,443	1,091	31.7%	0.61#	0.54–0.70	0.63#	0.55–0.72
35–44 years			2,986	846	28.3%	0.52#	0.46–0.60	0.53#	0.46–0.61
45–54 years			1,838	470	25.6%	0.46#	0.39–0.53	0.45#	0.39–0.53
> =55 years			774	154	19.9%	0.33#	0.27–0.41	0.34#	0.28–0.42

^1^ *MSM* men who have sex with men

^2^ *IDU* persons who are intravenous drug users

^3^ *HET* persons with heterosexual contact

^°^95% CI: 95% Confidence interval

*OR: odds ratio

# *p*-value: *p* < =0.001

## *p*-value: *p* < =0.05

## Discussion

This was the first analysis in Germany on national level to determine the proportion of recent HIV infections among newly diagnosed HIV cases by using the BED-CEIA according to ECDC corrections. One-third of newly diagnosed HIV infections between 2008 and 2014 were recent infections within the six months before diagnosis. Overall, we found a slight but significant increasing trend of recent HIV infection between 2008 and 2014, particularly among MSM and HET. The highest proportion of recent HIV infections was found in MSM (35.0%) in contrast to a low proportion in HET (20.6%). Logistic regression identified that only the small group of female German IDU had a higher chance of being newly diagnosed and coincidentally infected recently with HIV than MSM.

The overall proportion of recent HIV infections in Germany is similar to that of other European countries using the BED-CEIA for TRI. The overall proportion of recent HIV infections ranged from 23% among newly diagnosed cases in Catalonia, Spain (2006–2008) [32] to 35% in Sweden (2003–2010) [33]. However, in countries using other TRI, such as England, Northern Ireland, Wales or France, the proportion of recent HIV infections ranged between 15% [34] and 25% [35], which is lower than in those countries using the BED-CEIA for TRI. The proportion of newly diagnosed and recently infected cases is constantly low over the time compared to the high proportion of long standing and late presenting HIV infections. This is alarming from the perspective of prevention, because those who are diagnosed late are often unaware of their HIV infection for a long time and could therefore potently transmit HIV. As a consequence, early detection and diagnosis of HIV infection is of utmost importance to prevent new infections. As a strategy for eliminating infections, testing must be brought to scale, followed by immediate sustainable treatment, irrespective of the immune status.

Men who have sex with men (MSM) are the group most at risk for HIV infection in the European Union (EU)/European Economic Association (EEA), as well as in the United States, despite targeted prevention programmes since the beginning of the HIV epidemic [36]. MSM are also the group in Germany with the highest proportion of newly diagnosed cases of recent HIV infection, similar to other European countries which used the BED-CEIA [32, 33] or other serological tests [34, 37].

It is recommended that persons at risk for HIV infection - such as MSM - should be tested at least once a year or even more often, depending on their risk behaviour [38, 39]. This may result in a higher proportion of recently diagnosed HIV infections. It is known that the awareness, knowledge and perceived level of personal risk for HIV infection among MSM is high, which might be one reason for regular test seeking and the high proportion of recent HIV infection we found in this group. Even though they test more regularly than do other groups with risky behaviour, the proportion of recent HIV infections among MSM should be much higher. In a recent German MSM online survey, approximately 60% of the questioned MSM were tested within the previous 12 months, and approximately 53% said they are getting tested on a regular basis [40]. However, nearly two-thirds (65%) of MSM who were newly diagnosed with HIV were diagnosed later than 6 months after the infection occurred. Reasons for late diagnosis may vary among different groups. The fear of stigmatization, a large geographical distance to the next anonymous testing site, or a lack of risk awareness may be related to HIV testing behaviour, especially among MSM [40]. The lack of risk awareness might also be the reason for the low proportion of recent HIV infections among HET. To improve testing frequency in groups with risky behaviour, such as MSM, IDU or HET, prevention programmes should include the importance of regular and easy access to HIV testing sites and increase the awareness of risks.

Female intravenous drug users also showed a high proportion of newly diagnosed cases with recent HIV infection. We also found that female drug users of German origin had a higher chance than MSM for having a recent HIV infection. The high proportion of recent HIV infections might be related to the fact that women more often seek contact with the health care system or are better reached by testing offers for HIV or Hepatitis C during opioid substitution therapy. However, as the numbers in this sub-group were small due to the small group size, we believe that the result of the logistic regression may be related to statistical chance, and the associated results must be interpreted carefully and should be further observed in the following years. Nevertheless, this result shows the importance of adapted prevention programmes. All persons with risky sexual behaviour should be offered an HIV test regularly, whenever they have contact with the health care system. However, an HIV test should not only be offered by contact with the health care system, e.g. HIV/STI outreach clinics, it also should be included in harm reduction programmes for drug users and offered in low threshold drug services. Despite the continuous roll-out of HIV testing campaigns, services still need to improve the offers and the facilities for HIV testing to reduce late presentation.

The proportion of recent HIV infections decreased with age, meaning that long-standing infections were found more often in older age groups. This trend was also observed in other studies [33, 34, 37, 41]. The time between infection and diagnosis is usually shorter in younger persons; nevertheless, the proportion of recent HIV infection in persons older than 55 years is still nearly 24%, indicating that there is ongoing HIV-transmission in older age groups.

Persons who originated from abroad more often had a long-standing infection than did German natives. This could mean that most of these cases were infected and potentially diagnosed in their home country, but the cases were also diagnosed in Germany for the first time and thus reported by the laboratories as newly diagnosed in Germany. Another explanation might be that these people did not know about their infection and that they had not been reached appropriately by prevention programmes and testing strategies in Germany. To prevent HIV infections among persons from abroad, the prevention programmes have to be tailored in a way to reach those hard-to-reach groups. To improve understanding regarding persons originating from sub-Saharan Africa and living in Germany, a study was established in 2014 at the RKI that aimed to identify their specific needs and their knowledge about HIV in order to address sustainable prevention in this sub-group in Germany [42].

The proportion of recent infections among newly diagnosed cases increased over the time period. This could suggest a real increase in HIV infections. However, this increase is not reflected by the total number of newly diagnosed infections, which remained fairly stable over the past years [31]. Therefore, the increase in recent infections could reflect more specific testing during that time period. In Germany, data are only available regarding HIV diagnoses but not regrading negative test results or the total number of HIV tests performed. It is therefore impossible to draw firm conclusions on rates solely based on recency testing among newly diagnosed HIV cases without knowledge of the testing rates.

### Limitations

There are some limitations to our analysis. First, the interpretation of the proportion of recent infections among newly diagnosed HIV cases depends on testing patterns and the number of persons tested for HIV. There is only limited information available about the total number of persons tested in Germany. A study among HIV diagnostic laboratories estimated that approximately 1,6 million HIV screening tests were performed in 2011 [43]. In that study, the participating laboratories could not distinguish between performed tests and persons screened, and the study only had information about performed tests for one year. Interpreting time trends for HIV recency in Germany is therefore difficult. The HIV test-seeking pattern might vary between different populations; for example, MSM are tested more regularly and more frequently than other populations. Therefore, a direct estimation of the HIV incidence is not possible, and we can only report the proportion of recent infections among newly diagnosed sub-groups over time.

Second, the sensitivity and specificity of the BED-CEIA (81.7%; 89.1%) [15] are quite low, while the reported false recent rates (FRR) are high (7.4%) depending on the HIV-subtype distribution in the respective countries [44]. However, the BED-CEIA was evaluated using specimens from the German HIV-seroconverter cohort, where the date of infection was well-defined based on the laboratory diagnosis [29]. Therefore, the application of the BED assay to the German surveillance system is assumed to be valid to report the proportion of recent HIV infections among newly diagnosed HIV cases in Germany. Recently, we adapted the validation panel to an updated distribution of HIV-subtypes in Germany in order to re-calculate the FRR induced by the BED-CEIA, resulting in an FRR of 10% for Germany [45]. Because of the rather high FRR, a testing algorithm was applied using the recommendation of the ECDC [30]. For the reason that CD4 cell counts and viral load measurements are incomplete in the HIV notification system and are only reported in one-third of the notified cases, the recommended algorithm could only be used with the information about AIDS status. Therefore, the reported recent HIV infections may still be overestimated. Data to correct false long-standing test results are not available in the German HIV surveillance system; therefore, a correction is not possible. This might introduce an error into the data, because we might overestimate cases with long-standing infection. In 2016, we implemented a multi-assay algorithm, including the BED-CEIA and an avidity assay, to address this limitation.

## Conclusions

In conclusion, the surveillance of recent HIV infections is a useful additional tool to monitor the HIV epidemic in Germany. It allows the observation of ongoing HIV transmission in Germany, and we identified (sub) populations at risk who can now be addressed more specifically in prevention programs, such as offering more frequent HIV-testing to those populations at risk. We could also identify factors associated with recent HIV infection, such as age, transmission group and origin. An early diagnosis is important for preventing new infections. Furthermore, the information gained through this part of the HIV surveillance programme can improve the statistical estimation models of the prevalence and incidence of HIV in Germany.

However, there is still the potential to improve the surveillance of recent HIV infections, for example, by implementing serological tests for recent infections with a higher sensitivity and specificity or to apply two serological tests in a multi-assay algorithm to achieve a more accurate proportion of recent HIV infections among the newly diagnosed HIV cases in Germany. It is also important to have a valid and accurate documentation of clinical data (such as CD4 count and virus load) to correct falsely identified recent or long-standing infections, as is recommended internationally. Additionally, to calculate the HIV incidence in Germany, it is important to include the total number of persons tested for HIV in Germany into the surveillance of recent HIV infections.

## Abbreviations

95% CI: 95% confidence interval
AIDS: acquired immunodeficiency syndrome
BED-CEIA: BED-capture-enzyme immunoassay
CDC: Centers for Disease Control and Prevention
DPS: dried plasma spots
DSS: dried serum spots
ECDC: European Centre for Disease Prevention and Control
EEA: European Economic Association
EU: European Union
FLS: false long-standing
FRR: false recent rate
HET: persons with heterosexual contacts
HIV: human immunodeficiency virus
IDU: persons who are intravenous drug users
MSM: men who have sex with men
OR: odds ratio
RITA: recent test algorithm
RKI: Robert Koch- Institute
TRI: test for recent (HIV) infections
